# Caloric Restriction Attenuates Gentamicin-Induced Acute Kidney Injury and Is Associated with Changes in Oxidative Stress and Mitochondrial DNA Damage

**DOI:** 10.3390/antiox15060653

**Published:** 2026-05-22

**Authors:** Xinyu Liao, Nadezda V. Andrianova, Ljubava D. Zorova, Irina S. Sadovnikova, Dmitry S. Semenovich, Vasily N. Manskikh, Irina B. Pevzner, Artem P. Gureev, Egor Y. Plotnikov

**Affiliations:** 1 A. N. Belozersky Institute of Physico-Chemical Biology, Moscow State University, 119992 Moscow, Russia; starrain524@gmail.com (X.L.); andrianova@belozersky.msu.ru (N.V.A.); 7emenovich@gmail.com (D.S.S.); pevzner_ib@belozersky.msu.ru (I.B.P.); 2 V. I. Kulakov National Medical Research Center for Obstetrics, Gynecology and Perinatology, Ministry of Healthcare of Russian Federation, 117198 Moscow, Russia; 3 Department of Genetics, Cytology and Bioengineering, Voronezh State University, 394018 Voronezh, Russia

**Keywords:** dietary restriction, acute kidney injury, drug-induced nephrotoxicity, oxidative stress, mitochondria, autophagy, inflammation

## Abstract

Caloric restriction (CR) is known to activate a broad spectrum of cytoprotective signaling pathways and enhance tissue tolerance to various stressors, including those associated with the cytotoxic effects of pharmaceutical agents. Nephrotoxic drugs, such as aminoglycoside antibiotics, remain a major clinical concern due to their frequent use and potential to cause acute kidney injury (AKI), for which effective preventive strategies are still limited. In this study, we investigated whether CR applied for 5 weeks (4-week pretreatment + 1-week concurrent with AKI induction) can alleviate AKI triggered by the antibiotic gentamicin, with a focus on evaluating changes in antioxidant-related parameters and autophagy-associated signaling during CR-mediated nephroprotection. CR’s nephroprotective effects were evaluated using diagnostic assays, Western blotting, and histological analysis. Additionally, oxidative stress markers and mitochondrial integrity were assessed to analyze the impact of CR on antioxidant-related pathways. CR significantly improved renal function and structure, with reduced kidney injury markers (KIM-1, NGAL) and alleviated histological damage. Critically, CR mitigated oxidative stress, evidenced by decreased thiobarbituric acid reactive substances (TBARS) and protein carbonylation, as well as increased levels of the reduced form of glutathione and activity of glutathione peroxidase (GPx). A lowered Bcl-X_L_/X_S_ ratio was consistent with reduced apoptotic signaling, while reduced leukocyte infiltration reflected attenuated renal inflammation. Additionally, a reduction in mitochondrial DNA (mtDNA) lesions suggested that CR was associated with modulation of mitochondrial and metabolism-related pathways, with concurrent improvements in mitochondrial stability. Our findings demonstrate that CR attenuated gentamicin-induced AKI and was associated with changes in antioxidant-related parameters, reduced mtDNA damage, a decrease in inflammatory cell infiltration, and modulation of autophagy-related signaling.

## 1. Introduction

Acute kidney injury (AKI) remains a critical clinical challenge, particularly in high-risk populations exposed to nephrotoxic agents such as aminoglycoside antibiotics [1]. Among these, gentamicin, a cornerstone therapy for Gram-negative infections, induces AKI in 20–33% of drug-related cases [2], with clinical studies reporting AKI incidence in 24.4% of treated patients [3] and a statistically significant correlation with renal dysfunction in large cohorts [4]. Gentamicin-induced kidney damage involves a complex set of interrelated processes, including oxidative stress, mitochondrial dysfunction, and activation of inflammatory responses in tubular epithelial cells [5,6,7]. Autophagy disorders have also been described in this model, but their contribution to the progression of damage and repair processes remains poorly defined. Together, these mechanisms are considered key factors contributing to the structural and functional damage to renal tissue.

Caloric restriction (CR) is considered a potential non-pharmacological approach that can alleviate the course of such injuries [8,9,10]. Although most research focuses on its systemic metabolic effects, such as its impact on body weight [11,12] and life expectancy [13,14], there has been growing interest in studying CR in the context of acute pathological conditions. In particular, CR has shown a protective effect in models of renal ischemia–reperfusion [15,16], as well as in AKI caused by antineoplastic drug (e.g., cisplatin) administration [17,18], suggesting the possible relevance of this approach for antibiotic-induced renal injury, given the partial overlap of key pathogenetic mechanisms.

CR is known to improve cellular redox balance [19]; however, it remains unclear to what extent these effects occur directly in renal tissue during gentamicin-induced damage, where oxidative stress plays an important role [20,21]. Although a decrease in oxidative stress with CR has been demonstrated, for example, in diabetic conditions [22,23,24], its effect on antioxidant and redox-buffer systems in this model remains insufficiently characterized. Similarly, despite evidence of mitochondrial DNA disruption and altered mitochondrial dynamics under gentamicin exposure [5,25,26], the potential association between the protective effects of CR and processes related to mitochondrial damage in AKI requires further investigation.

Additionally, CR can induce autophagy, a process involved in the removal of damaged proteins and organelles [27,28,29]. However, the contribution of autophagy to the development and limitation of gentamicin-induced kidney damage remains poorly understood, and data from other models (such as ischemia/reperfusion) suggest a possible context-dependent nature of its effects. Therefore, it is of interest to assess whether changes in autophagy markers are associated with other physiological and signaling changes in this damage model. Thus, despite available data on the systemic effects of CR and its protective effect in selected models of renal injury, its role in gentamicin-induced nephrotoxicity, as well as its association with key pathogenetic processes, including oxidative stress, inflammation, and signaling pathways related to mTORC1 and autophagy, remains poorly defined.

The present study was designed to evaluate whether CR (4 weeks of pretreatment followed by 1 week during gentamicin exposure) attenuates renal injury in a model of drug-induced AKI. To this end, we assessed renal function, histological changes, and a set of molecular markers related to oxidative stress, inflammation, and autophagy-associated signaling. This approach was intended to determine whether CR-induced renoprotection is associated with modulation of these pathways, rather than to establish a definitive causal mechanism.

## 2. Materials and Methods

### 2.1. Animals

This study was performed on male Wistar rats aged 3 months, with an average weight of 330–350 g at the start of the experiment. The rats were acclimatized to laboratory conditions for 2 weeks prior to the experiment, with free access to water and standard rat chow. The animals were randomly divided into 4 groups: (1) *ad libitum* (“AL”, *n* = 4), (2) caloric restriction (“CR”, *n* = 4), (3) *ad libitum* + gentamicin (“AL + GM”, *n* = 8), and (4) caloric restriction + gentamicin (“CR + GM”, *n* = 8).

The animal protocols were reviewed and approved by the Animal Ethics Committee of the A.N. Belozersky Institute of Physico-Chemical Biology Lomonosov Moscow State University (Protocol 006-1/2/2024 from 1 February 2024). All procedures were performed in accordance with the “Animal Research: Reporting of In Vivo Experiments” (ARRIVE) guidelines. Animals had unlimited access to water and were maintained in cages in a temperature-controlled environment (20 ± 1 °C) under a 12 h/12 h light/dark regime.

### 2.2. CR Protocol

**Figure 1:**
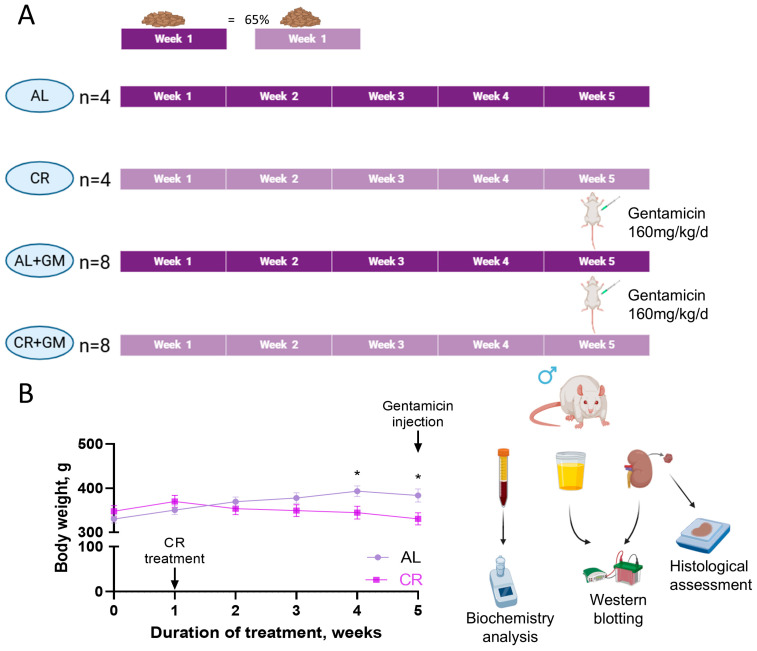
Experimental design of the study (**A**) and changes in body weight of rats during the experiment (**B**). * *p* < 0.05 between “AL” and “CR” groups in a specific week of the experiment. AL—*ad libitum*; CR—caloric restriction; GM—gentamicin. Created in BioRender. Buyan, M. (2026), https://BioRender.com/selwamr (accessed on 10 April 2026).

During a 2-week acclimatization period, food intake was registered daily for all rats. Then rats in the “CR” or “CR + GM” groups started to receive 35% CR, so rats were provided with 65% of the mean daily food intake. We restricted total food intake, including micronutrients, and no additional micronutrients were supplemented in the “CR” group. This dietary regimen was maintained for 5 weeks total: 4 weeks of 35% CR pretreatment and 1 week of the same diet concurrently with or without gentamicin administration (Figure 1). Rats from the “AL” and “AL + GM” groups had unlimited access to food throughout the whole experiment. Rats subjected to CR exhibited a significant decrease in body weight compared to the “AL” group, with differences of 12.3% at the 4th week and 13.7% at the 5th week (Figure 1B). 

### 2.3. AKI Modeling

After 4 weeks of 35% CR pretreatment, AKI was induced in the “AL + GM” and “CR + GM” groups by administering gentamicin (“Mosagrogen”, Moscow, Russia) at a dose of 160 mg/kg/day i.p. for 6 consecutive days. This dose was chosen based on previous studies to ensure the development of AKI [6,30]. Blood, urine, and kidney samples were collected from rats in all groups on day 7 for further analysis.

### 2.4. Western Blotting

Urine samples were subjected to centrifugation at 10,000× *g* for 5 min and then mixed with an equal volume of 2× sample buffer containing 10% 2-mercaptoethanol, followed by boiling for 5 min. Kidney tissues were homogenized in phosphate-buffered saline (PBS) containing 1 mM protease inhibitor phenylmethylsulfonyl fluoride (PMSF), and subsequently centrifuged at 1000× *g* for 3 min. Protein concentration was determined in kidney samples using the bicinchoninic acid assay kit (Sigma-Aldrich, St. Louis, MO, USA). Prior to gel electrophoresis, samples were centrifuged again at 10,000× *g* for 5 min. For urine samples, 20 μL of each sample was loaded onto 15% Tris-glycine polyacrylamide gels, while for kidney samples, 10 μg of protein was loaded per lane. Following electrophoretic separation, proteins were transferred onto PVDF membranes (Amersham Pharmacia Biotech, Amersham, UK). Membranes were blocked with 5% non-fat milk in PBS containing 0.05% Tween-20 and then incubated with the primary antibodies anti-p70 S6 kinase (p-p70 S6K) 1:1000 rabbit (#9202S, Cell Signaling, Boston, MA, USA), anti-LC3-A/B 1:1000 rabbit (#12471, Cell Signaling, USA), anti-KIM-1 1:1000 mouse (#MAA785Ra21, Cloud Clone Corp., Wuhan, China), anti-NGAL 1:1000 rabbit (#AB63929, Abcam, Cambridge, MA, USA), anti-proliferating cell nuclear antigen (PCNA) 1:1000 rabbit (#13110, Cell Signaling, USA), anti-myeloperoxidase (MPO) 1:1000 rabbit (#PAA601RA01, Cell Signaling, USA), anti-CD45 1:1000 rabbit (#PAB030Ra01, Cell Signaling, USA), anti-CD68 1:1000 rabbit (#DF7518, Cell Signaling, USA), anti-CD86 1:1000 rabbit (#SAB5700710, Sigma, USA), anti-Bcl-X 1:1000 rabbit (#2764, Cell Signaling, USA), and anti-β-actin 1:2000 mouse (#A2228, Sigma-Aldrich, St. Louis, MO, USA), overnight at 4 °C. Subsequently, membranes were incubated with horseradish peroxidase-conjugated secondary anti-rabbit or anti-mouse antibodies (Jackson ImmunoResearch, West Grove, PA, USA) and probed with the Advansta Western Bright ECL kit (Advansta, San Jose, CA, USA). Carbonylated proteins were measured using the OxyBlot kit according to the manufacturer’s instructions (S7150 OxyBlot Protein Oxidation Detection Kit, Millipore, Temecula, CA, USA). Protein bands were visualized using the V3 Western Blot Imager (Bio-Rad, Hercules, CA, USA). Western blot results for kidney homogenates were normalized to the intensities of β-actin of the corresponding samples. Densitometric analysis of Western blot bands was performed using ImageLab software (version 6.1.0, Bio-Rad, Hercules, CA, USA).

### 2.5. Biochemical Analysis of Serum

To confirm AKI, blood samples were taken 24 h after the last injection of gentamicin from the carotid artery to determine serum creatinine (SCr) and blood urea nitrogen (BUN). After 15 min of storage at room temperature, the clot was removed by centrifugation at 2000× *g* for 5 min. The resulting serum was frozen and later analyzed for SCr and BUN concentrations using the AU480 Chemistry System (Beckman Coulter Inc., Brea, CA, USA) according to the manufacturer’s instructions.

### 2.6. Histological Assessment

Kidneys were fixed in 4% paraformaldehyde for 24 h, dehydrated through a graded ethanol series, cleared in xylene, and embedded in paraffin. Sections (4 μm thick) were deparaffinized in xylene and rehydrated through a graded ethanol series to water. H&E staining was performed by immersing sections in Mayer’s hematoxylin for 5 min at room temperature, followed by differentiation in 1% acid alcohol and bluing in running tap water for 5 min. Sections were then stained with 1% eosin B for 10 min. All sections were dehydrated through graded alcohols, cleared in xylene, and mounted with neutral balsam [31,32]. The stained sections were examined using an Axio Scope A1 microscope (Carl Zeiss, Oberkochen, Germany) equipped with an MRc.5 camera (Carl Zeiss, Germany).

### 2.7. Determination of Oxidative Stress 

The content of TBARS in kidney homogenate was determined after incubation with 50 μM Mohr’s salt and 0.5 mM ascorbate at 37 °C for 1 h. Samples were boiled with TBA reagent, cooled, and centrifuged at 3000× *g* for 10 min. The absorbance at 532 nm was determined in the supernatant. The TBARS content was estimated from an extinction coefficient of 156 mM^−1^ cm^−1^ [33]. The level of reduced glutathione (GSH) in kidney tissue was determined using the Ellman’s reagent by measuring absorbance at 412 nm. GSH content was estimated using an extinction coefficient of 14.150 mM^−1^ cm^−1^ [34]. Values were normalized to the total protein content in kidney tissue homogenates, determined by the Lowry method. 

The activity of glutathione peroxidase (GPx, EC 1.11.1.9) was determined via a spectrophotometric kinetic method using tert-butyl hydroperoxide as substrate [35]. Measurements were performed at 340 nm on a Zenyth 3100 multimodal plate reader (Anthos Labtec, Salzburg, Austria) at 37 °C.

Catalase activity (EC 1.11.1.6) was determined using a spectrophotometric method based on the interaction of excess hydrogen peroxide with ammonium heptamolybdate [36]. Measurements were carried out at a wavelength of 405 nm on an Ekros PE-5400UV spectrophotometer (Ekrokhim, Saint Petersburg, Russia), and 1 nmol of H_2_O_2_ consumed in 1 min at 37 °C was taken as 1 unit (U) of catalase activity.

### 2.8. Assessment of mtDNA Copy Number

Relative mitochondrial DNA (mtDNA) copy number was assessed by qPCR using a 5× qPCRmix-HS SYBR mixture (Evrogen, Moscow, Russia). To amplify rat mtDNA, the following pair of primers was used: forward: 5′-CTCAAAGGACTTGGCGGTACT-3′; reverse: 5′-GCTGAATTAGCGAGAAGGGGT-3′. The obtained results were normalized to the *Gapdh* gene (Table 1) in nuclear DNA. Reaction conditions: denaturation at 95 °C for 3 min; 35 cycles of denaturation at 95 °C for 10 s, primer annealing at 59 °C for 30 s, and elongation at 72 °C for 30 s. mtDNA copy number was calculated with the Bio-Rad CFX Manager software (version 2.1) using the formula 2^−ΔΔCq^.

### 2.9. Estimation of mtDNA Damage

The amount of mtDNA damage was assessed by PCR of long fragments using Encyclo polymerase (Evrogen, Russia). The protocol and primer panels for rat mtDNA were developed and optimized previously [37]. The method is based on the assumption that the presence of damage in the DNA structure, e.g., single-strand breaks, modified bases or their adducts, impairs the function of DNA polymerase and the accumulation of the PCR product. Therefore, the efficiency of amplification of a DNA region is inversely proportional to the number of DNA molecules with damage. Reaction conditions: general denaturation at 95 °C for 3 min; then 35 cycles of denaturation at 95 °C for 10 s, primer annealing at 59 °C for 4 min 30 s, and elongation at 72 °C for 30 s. The obtained ΔCq values between the control and experimental (damaged) long fragments were compared with the ΔCq of the control and experimental short fragments, which were used as references.

### 2.10. Number of Large-Scale mtDNA Deletions

Total DNA from rat tissues was isolated using a PROBA-GS kit (DNA-technology, Russia) according to the protocol recommended by the manufacturer. The relative number of large-scale deletions in rat mtDNA (ΔmtDNA4834) was determined by qPCR using TaqMan probes as described previously [38]. The amount of ΔmtDNA4834 (a region formed by the joined sequences flanking the deleted mtDNA region) was normalized to the total amount of mtDNA. To normalize the number of deletions to the number of mtDNA copies, the D-loop region was amplified using the 5× qPCRmix-HS mixture (Evrogen, Russia). Reaction conditions: denaturation at 95 °C for 3 min; 35 cycles of denaturation at 95 °C for 10 s, primer annealing at 64 °C for 15 s, and elongation at 72 °C for 20 s.

### 2.11. Quantitative PCR

To obtain cDNA on the RNA matrix, the RIVERTA-L kit (AmpliSens, Moscow, Russia) was used. The reaction was performed according to the protocol on a BIS M 111-02-48 amplifier (NovosibBioPribor, Novosibirsk, Russia). Quantitative PCR analysis was performed on a CFX96TM Real-Time System thermocycler (Bio-Rad, Hercules, CA, USA) using a qPCRmix-HS SYBR kit (Evrogen, Moscow, Russia). PCR conditions were as follows: total denaturation in 5 min at 95 °C, and then 45 cycles of 10 s at 95 °C, 30 s at 59 °C, and 30 s at 72 °C. The normalized expression level was calculated using the formula 2^−ΔΔCq^. The glyceraldehyde 3-phosphate dehydrogenase gene (*Gapdh*) was used as a reference. The primer sequences of the genes studied are listed in Table 1.

### 2.12. Statistical Analysis

Power analysis was performed using STATISTICA 10 software (StatSoft, Inc., Tulsa, OK, USA) with a significance level of 0.05. A priori power analysis indicated that the selected sample sizes provided statistical power ≥0.8 for detection of biologically relevant effect sizes at α = 0.05, based on variability observed in previous experiments using similar AKI models and endpoints. Data were presented as the mean ± standard error of the mean (SEM). The data were tested for normality using the Shapiro–Wilk test. For comparison of two groups, the *t*-test was used in the case of parametric variables and the Mann–Whitney U-test in the case of non-parametric variables. For comparison among 3 or more groups, one-way ANOVA with Tukey’s post hoc test or the Kruskal–Wallis test with Dunn’s test in the case of non-normally distributed data were used. Data was analyzed using Microsoft Excel software (version KB4011684, Redmond, WA, USA) and GraphPad Prism (version 8, GraphPad Software Inc., La Jolla, CA, USA). 

## 3. Results

### 3.1. Effects of CR on Markers of Autophagy, Mitochondrial DNA and Oxidative Stress in the Kidney

**Figure 2:**
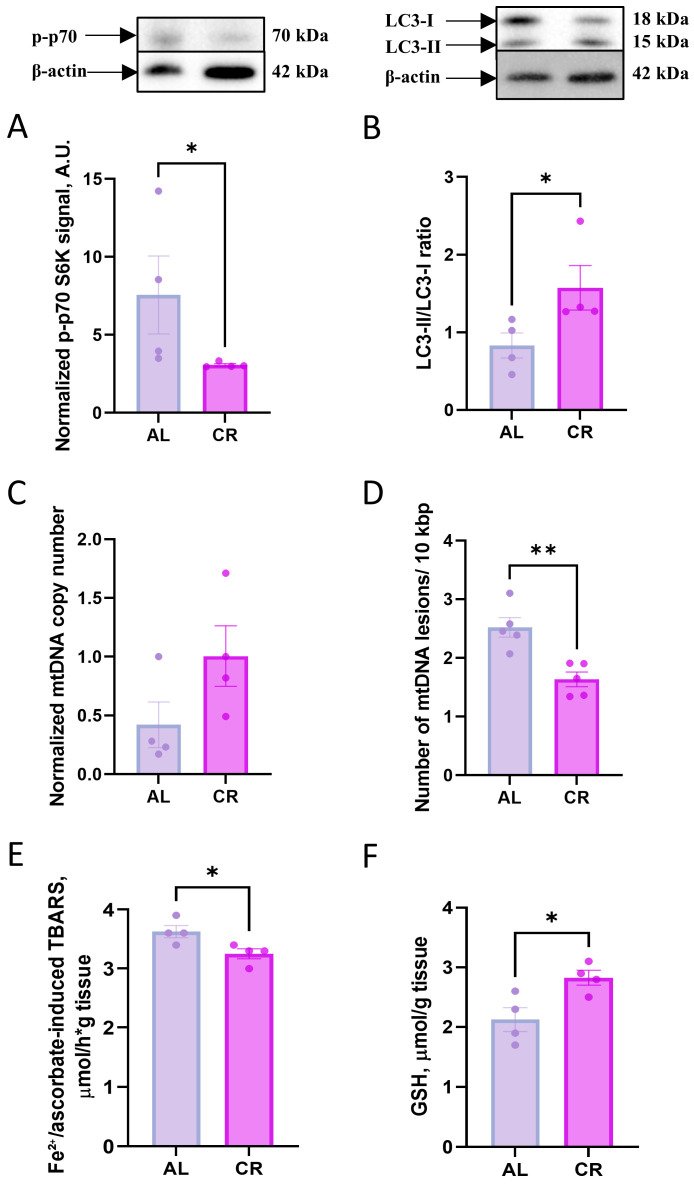
Assessment of markers of mitochondrial function and oxidative stress markers in kidney tissue after 5-week CR treatment. (**A**) p-p70 S6K level; (**B**) LC3-II/LC3-I ratio; (**C**) copy number of mtDNA; (**D**) mtDNA lesion number; (**E**) Fe^2+^/ascorbate-induced TBARS levels; (**F**) GSH levels. * *p* < 0.05, ** *p* < 0.01 compared to the AL group. Individual data points are shown. AL—*ad libitum*; CR—caloric restriction.

First, we evaluated whether the 5-week CR protocol affects molecular markers of autophagy and mTORC1 signaling in the kidneys in the absence of gentamicin-induced damage. CR led to a decrease in phosphorylation of p70 S6 kinase compared with the *ad libitum* control group (Figure 2A), reflecting the inhibition of mTORC1. At the same time, the LC3-II/LC3-I ratio was increased in the “CR” group (Figure 2B).

Next, we assessed parameters related to mitochondrial quality control, primarily mtDNA content and the accumulation of damaged regions. The number of mtDNA copies did not differ significantly between the “CR” and “AL” groups (Figure 2C), while the frequency of mtDNA damage was lower in the “CR” group (Figure 2D) compared to the “AL” group. Since mitochondrial dysfunction often leads to oxidative stress, we evaluated redox-buffer system parameters in kidney cells, such as glutathione levels and resistance to lipid peroxidation. After ex vivo treatment with Fe^2+^/ascorbate, lower levels of TBARS were found in kidney homogenates from CR animals compared with those in the “AL” group (Figure 2E). In addition, CR was associated with an increase in reduced GSH levels in renal tissue (Figure 2F). Together, these results indicate that 5 weeks of CR leads to changes in markers associated with mTORC1 signaling, autophagy, mtDNA damage, and modulation of redox status, contributing to higher resistance of kidney tissue to oxidative stress.

### 3.2. Effects of CR on AKI Severity

In the next step, we evaluated the effect of CR on renal impairment in a model of gentamicin-induced nephropathy. Rats in the “AL + GM” and “CR + GM” groups were injected with gentamicin (160 mg/kg/day for 6 days). Measurement of SCr and BUN levels showed impaired renal function in “AL + GM” rats. The SCr level increased 6.4-fold compared to the AL control (AL: 53.0 ± 5.5 µM vs. AL + GM: 341.1 ± 169.4 µM), and the BUN level increased 4.8-fold (AL: 8.9 ± 1.6 mM vs. AL + GM: 42.8 ± 10.2 mM). In the “CR + GM” group, SCr levels were comparable to those in the AL control (55.5 ± 15.1 µM), and BUN levels were 81% lower than in the “AL + GM” group (8.0 ± 3.3 mM).

**Figure 3:**
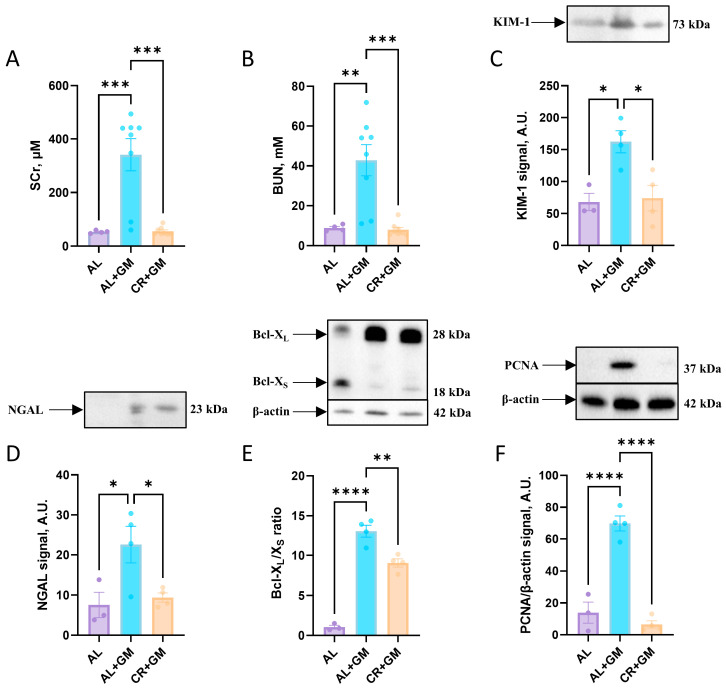
Kidney function and injury markers during gentamicin-induced AKI and CR treatment. The severity of AKI was evaluated by SCr (**A**) and BUN (**B**) concentrations in serum. Kidney tissue injury analyzed by urinary KIM-1 (**C**) and NGAL (**D**) levels. Bcl-X_L_/X_S_ ratio (**E**) and PCNA levels (**F**) in kidney tissue. * *p* < 0.05, ** *p* < 0.01, *** *p* < 0.001, **** *p* < 0.0001 compared to each group. Individual data points are shown. AL—*ad libitum*; AL + GM—*ad libitum* + gentamicin; CR + GM—caloric restriction + gentamicin.

We then evaluated early markers of tubule damage. The urinary KIM-1 level increased approximately 12-fold in “AL + GM” rats compared with the AL control, whereas in the “CR + GM” group it remained at baseline. The NGAL level increased almost 12-fold in the “AL + GM” group, while in the “CR + GM” group the values were comparable to those in the control. The ratio between pro- and anti-apoptotic forms of Bcl-X_L_/Bcl-X_S_ was evaluated. An increase in this ratio was observed in the kidneys of the “AL + GM” group compared with the “AL” group (Figure 3E). In the “CR + GM” group, the ratio was lower than in the “AL + GM” group and did not differ statistically from that in the “AL” group.

A quantitative determination of the PCNA tissue proliferation marker was also performed. In “AL + GM” rats, PCNA levels increased more than 5-fold compared with the AL control (Figure 3F). Only a slight increase was observed in the “CR + GM” group (Figure 3F), which is statistically equivalent to the values in the control group. These data show that the use of the CR protocol leads to preservation of renal function, decreased levels of tubule damage markers, changes in the ratio of apoptosis-associated proteins, and reduced proliferative activity in the gentamicin-induced nephropathy model.

We also measured the relative number of large-scale deletions in mtDNA obtained from kidney tissue of rats, as these deletions can indicate the organelle’s state and provide direct evidence of mitochondrial damage. We observed that the number of large-scale mtDNA deletions tended to increase by 55% after gentamicin administration in rats compared to the AL group; however, the observed changes did not reach statistical significance. The values of large-scale deletions in mtDNA in the kidneys of rats from the “CR + GM” group were similar to those of the control AL animals.

**Figure 4:**
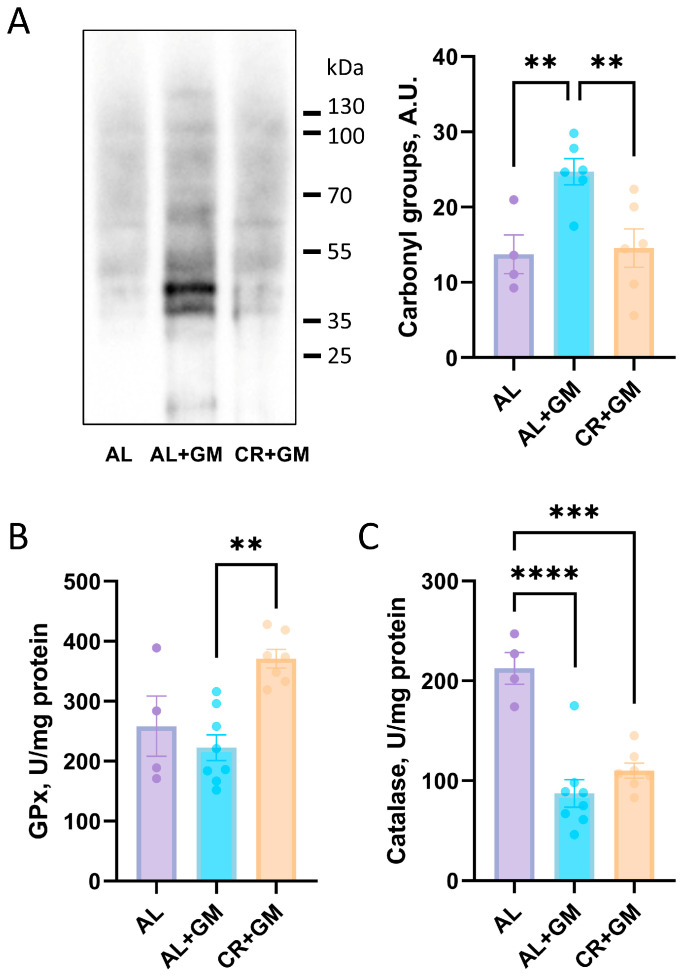
Assessment of oxidative stress markers in kidney tissue during gentamicin-induced AKI and CR treatment. (**A**) OxyBlot staining for carbonylated proteins; (**B**) GPx activity; (**C**) catalase activity. ** *p* < 0.01, *** *p* < 0.001, **** *p* < 0.0001 (one-way ANOVA with Tukey’s post hoc test). Individual data points are shown. AL—*ad libitum*; AL + GM—*ad libitum* + gentamicin; CR + GM—caloric restriction + gentamicin.

To further characterize oxidative damage in renal tissue under gentamicin-induced AKI and CR treatment, we assessed the level of carbonylated proteins and the activities of catalase and GPx, key antioxidant enzymes (Figure 4). Protein carbonylation is a widely used marker of oxidative stress, reflecting modifications generated by various free radicals and lipid peroxidation by-products. The level of carbonylated proteins increased after 6 days of gentamicin administration but was significantly lower in the “CR + GM” group (Figure 4A). After gentamicin administration, GPx activity was comparable to that in intact rats, whereas CR treatment significantly increased it compared to GM rats (Figure 4B), which may indicate enhanced protection against peroxides, including lipid hydroperoxides. At the same time, gentamicin treatment led to a significant decrease in catalase activity in kidney tissue, while CR partially attenuated this decrease, although the difference between the “AL + GM” and “CR + GM” groups did not reach statistical significance. These findings indicate that CR partially attenuates gentamicin-induced oxidative damage in kidney tissue and increases its tolerance to injury.

### 3.3. The Effect of CR on the Parameters of the Inflammatory Response

**Figure 5:**
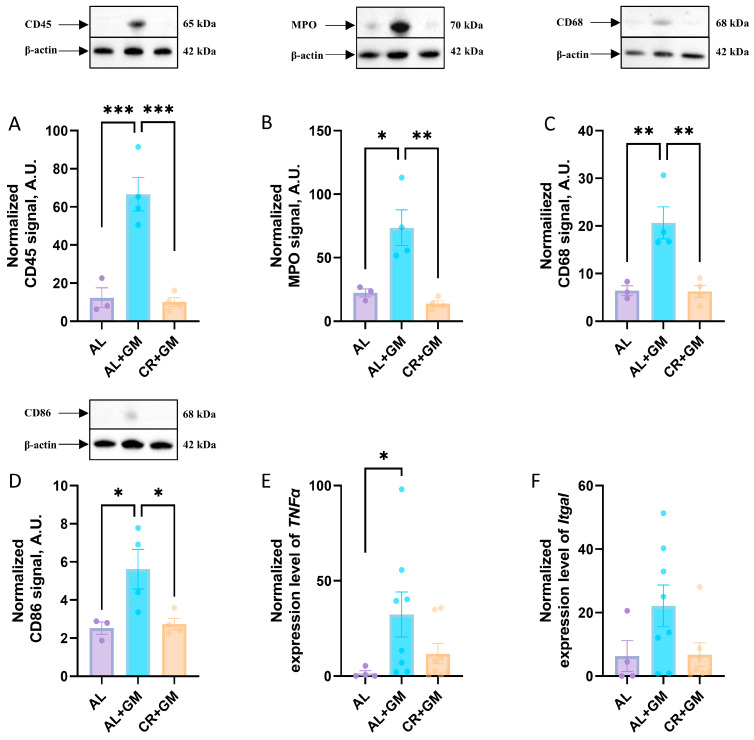
Comprehensive assessment of inflammatory response markers after 5-week CR treatment. Assessment of total leukocyte infiltration based on CD45 level (**A**) and MPO+-based evaluation of neutrophil infiltration (**B**) in kidney tissue. CD68^+^ macrophage (**C**) and CD86^+^ M1-macrophage marker (**D**) abundance. The expression of *TNFα* (**E**) and *Itgal* (**F**) in kidney tissue. * *p* < 0.05, ** *p* < 0.01, *** *p* < 0.001 compared to each group. Individual data points are shown. AL—*ad libitum*; AL + GM—*ad libitum* + gentamicin; CR + GM—caloric restriction + gentamicin.

We analyzed immune cell markers in kidney tissue. The CD45 level increased threefold in the “AL + GM” group compared with the “AL” control (Figure 5A). The CD45 level was lower in the “CR + GM” group than in the “AL + GM” group. The MPO level, an enzyme that drives both the oxidative burst and neutrophil chemotaxis [39], increased threefold in the “AL + GM” group compared to the control and was comparable to that in intact animals in the “CR + GM” group (Figure 5B). The CD68 level was more than four times higher in the “AL + GM” group (Figure 5C), but no such increase was observed in the “CR + GM” group. Gentamicin administration significantly increased CD86 levels in kidney tissue, and in the “CR + GM” group, its amount was reduced compared with “AL + GM” (Figure 5D). Expression of the proinflammatory cytokine *TNFα* (Figure 5E) and the neutrophilic infiltration marker *Itgal* (Figure 5F) increased in the “AL + GM” group, and in the “CR + GM” group, there was a downward trend in these markers. Thus, gentamicin treatment led to a significant increase in markers of infiltration of various types of leukocytes and production of proinflammatory cytokines, while CR reduced the severity of the inflammation.

### 3.4. Morphological Alterations in the Kidney After GM and CR Treatment

**Figure 6:**
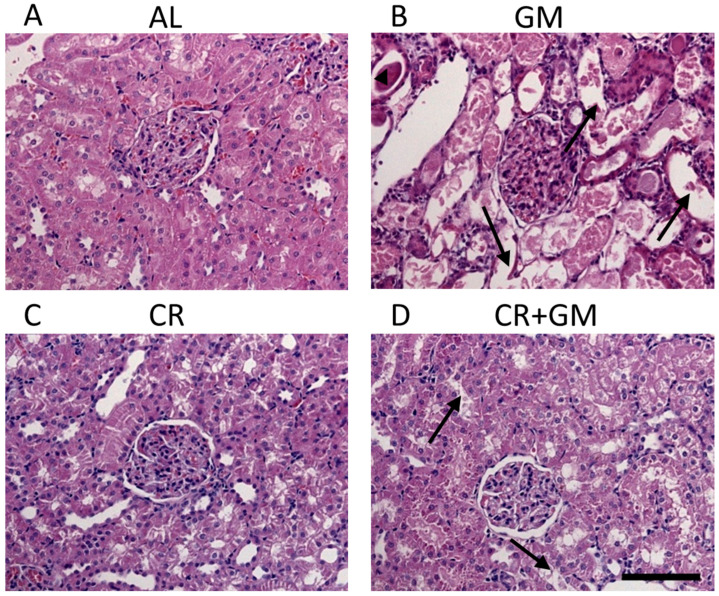
CR attenuates gentamicin-induced histopathological damage in rat renal tissue. Representative photomicrographs of kidney sections (H&E staining) from the experimental groups. (**A**) AL group with normal renal morphology, intact glomeruli and proximal tubules with preserved brush borders and nuclei. (**B**) GM group showing severe damage with widespread tubular damage, with loss of brush borders and nuclei (black arrows), and the presence of hyaline casts (black arrowhead) and cell debris. (**C**) CR group’s renal morphology is comparable to that of the AL group, with normal glomeruli and tubules. (**D**) “CR + GM” group with marked attenuation of gentamicin-induced damage. The severity is significantly reduced compared to the GM group, with fewer and smaller hyaline casts (black arrowhead). Scale bar: 100 µm (applies to all panels). Original magnification: 400×. AL—*ad libitum*; CR—caloric restriction; GM—gentamicin.

To evaluate structural protection by CR, we performed a blinded histopathologic analysis of kidney H&E-stained sections. The AL kidneys (Figure 6A) exhibited preserved renal architecture with intact glomeruli, normal tubular epithelium and continuous brush borders—consistent with baseline physiologic integrity. In contrast, AL + GM kidneys (Figure 6B) displayed severe damage: widespread tubular necrosis, characterized by nuclear pyknosis and loss of brush borders (black arrows), and karyolysis, desquamated cell debris, and abundant eosinophilic hyaline cast formation (black arrowhead), indicating gentamicin-induced tubular injury. It should be noted that no significant changes visible under light microscopy were found in the renal glomeruli.

CR treatment alone (Figure 6C) maintained near-normal morphology, with intact glomeruli and tubules indistinguishable from those in AL controls. Notably, CR kidneys exhibited mild cytoplasmic vacuolization in proximal tubules, a feature previously associated with adaptive metabolic reprogramming rather than injury. This observation aligns with our baseline data showing CR-induced autophagy signaling, where vacuoles may represent autophagosomes. Kidneys of rats from the “CR + GM” group demonstrated profound structural protection compared to those in “AL + GM” rats (Figure 6D). Tubule damage was focal and attenuated, with preserved brush borders in the majority of proximal tubules (black arrows), and the hyaline casts and cell debris were almost completely absent.

Overall, the CR protocol was associated with partial preservation of kidney histological structure and a reduction in morphological signs of tubular damage in the gentamicin-induced nephropathy model.

## 4. Discussion

The kidneys are highly dependent on mitochondrial function and tightly regulated redox homeostasis, making them particularly vulnerable to harmful effects that disrupt these processes, including drug toxicity [40,41]. AKI caused by gentamicin is known to lead to oxidative stress, inflammatory reactions [42], and alterations in cell quality control mechanisms [43,44], which contribute to structural [45] and functional [46] disorders of renal tissue.

In this study, we evaluated whether short-term CR can reduce gentamicin-induced kidney damage and whether this is related to changes in individual markers reflecting oxidative stress, inflammation, and mTORC1–autophagy signaling. Our results show that CR was associated with preservation of kidney function, decreases in the levels of damage biomarkers, and a reduction in histological changes. In addition, CR was associated with changes in markers related to redox status, mtDNA damage, and inflammatory cell infiltration. These data support the suggestion that CR may modulate several processes related to gentamicin-induced nephrotoxicity.

CR is an effective intervention known to attenuate age-related diseases [47], oxidative stress [48] and inflammation [49], thereby improving health and lifespan across species [50,51,52]. In the context of kidney health, chronic CR has been shown to delay the onset and progression of kidney disease and attenuate proteinuria, lipid deposition and fibrosis [16,53,54]. While these metabolic benefits, including improved autophagy, reduced oxidative damage and regulation of apoptosis, are well established in chronic models [11,55], the potential of short-term CR to protect against drug-induced AKI remains largely unexplored. Previous studies using longer-term CR regimens (≥8 weeks) have shown an improvement in mitochondrial function and a reduction in oxidative stress [56,57,58], but none have specifically addressed efficacy against gentamicin-induced nephrotoxicity, which is a common and serious clinical problem with limited effective treatments beyond kidney transplantation [59,60,61,62].

In our study, CR led to the expected reduction in body weight (Figure 1B), a hallmark of CR associated with metabolic remodeling [56,63,64]. Furthermore, we demonstrated that CR pretreatment significantly ameliorated renal dysfunction, as evidenced by reduced levels of conventional (BUN, SCr) and more sensitive (KIM-1, NGAL) AKI biomarkers (Figure 3A–D). The marker of apoptosis, i.e., the Bcl-X_L_/X_S_ ratio (Figure 3E), and downregulation of PCNA (Figure 3F) also indicate a state of reduced cellular damage and diminished need for proliferative repair. Alterations in the Bcl-X_L_/Bcl-X_S_ ratio may reflect changes in apoptosis-associated signaling. Members of the Bcl-2 family are key regulators of mitochondrial apoptotic pathways, and shifts in the balance between anti-apoptotic and pro-apoptotic isoforms influence cell survival [65,66]. Apoptotic processes are also closely linked to inflammatory responses, as cell death can modulate immune activation through the release of mediators such as DAMPs from injured or dying cells [67,68]. Therefore, the observed changes in the Bcl-X_L_/Bcl-X_S_ ratio may be associated not only with altered susceptibility of renal cells to stress-induced damage but also with modulation of inflammation in the kidney.

The involvement of the mTORC1 signaling system and autophagy in the observed effects of CR is one possible mechanism underlying the identified changes. In our study, CR was accompanied by a decrease in p70 S6K phosphorylation and an increase in the LC3-II/LC3-I ratio in renal tissue. These changes are usually considered indicators of a decrease in mTORC1 activity and alterations in processes associated with autophagy [69]. Autophagy is considered an important component of cellular homeostasis, especially under stress, when it promotes the renewal of damaged proteins and organelles [43,44]. In the context of kidney damage, both protective and detrimental functions of autophagy have been described, depending on the situation. However, in this study, we assessed only static markers of autophagy and therefore cannot distinguish between increased autophagosome formation and altered autophagic flux.

Given these limitations, changes in LC3 levels and mTORC1 signaling pathways should be interpreted with caution. Although they are consistent with the modulation of CR pathways associated with autophagy, they do not provide direct evidence of increased autophagic activity or its causal role in the observed nephroprotective effects. Nevertheless, the association between these markers and reduced kidney damage suggests that modulation of autophagy mechanisms and mTOR/AMPK signaling pathways and quality control may contribute to the overall protective phenotype in the kidney.

Although we cannot unequivocally determine the potential contribution of autophagy to the regulation of inflammation in the gentamicin nephrotoxicity model, the association of CR with positive changes in several proinflammatory parameters in the kidney appears quite evident, at least at the phenomenological level. Specifically, pretreatment with CR was accompanied by a marked decrease in the infiltration of CD45+, CD68+, CD86+, and MPO+ immune cells (Figure 5A–D), indicating a reduction in the inflammatory response. This aligns with previously described anti-inflammatory effects of CR [29]. Notably, the expression of *TNFa* and *Itgal* remained elevated, although there was a tendency for them to decrease (Figure 5E,F), which may suggest a differential effect of CR on leukocyte infiltration (CD45+, CD68+ and CD86+) rather than global suppression of proinflammatory cytokines (e.g., *TNFα*, *Itgal*).

In this context, it can be assumed that CR predominantly affects leukocyte infiltration rather than uniformly suppressing proinflammatory signaling. This interpretation is consistent with data describing the selective modulation of tissue inflammation compared with systemic inflammation in short-term CR [70]. Additionally, given the well-established role of mTORC1 in regulating inflammatory reactions [71], the observed changes may be related to modulation of this pathway. However, our data do not allow us to draw direct conclusions about the causal relationships between mTORC1 signaling, autophagy, and immune regulation.

Our study also observed the effect of CR on changes in markers associated with oxidative stress and mitochondrial integrity. Specifically, CR was accompanied by increased tissue tolerance to ex vivo-induced oxidative stress [72] and higher levels of reduced glutathione in kidney tissue. Furthermore, the increase in GSH is consistent with previous reports describing possible involvement of Nrf2-related antioxidant responses during CR [73], since Nrf2 is a known regulator of antioxidant enzyme expression [74]. Beyond Nrf2, CR may concurrently activate the FoxO family of transcription factors [75], fortifying the antioxidant defense network [76]. Upon activation, nuclear FoxO promotes the expression of a distinct set of antioxidant genes, such as manganese superoxide dismutase and catalase [76], complementing the Nrf2-mediated regulation of GSH and thioredoxin systems by targeting mitochondrial and peroxisomal ROS [77]. Thus, CR-associated changes in redox parameters may involve coordinated regulation of pathways linked to Nrf2 and FoxO signaling, both of which have previously been implicated in antioxidant responses during CR diets.

Additionally, there was a decrease in the frequency of mtDNA damage, while the number of mtDNA copies remained unchanged. These findings are consistent with changes in the redox state of renal tissue under CR. Although we did not directly measure the production of reactive oxygen species or the activity of antioxidant pathways in vivo, the observed differences in TBARS and glutathione levels should be interpreted as changes in parameters related to resistance to oxidative stress in gentamicin nephrotoxicity.

Mitochondrial dysfunction caused by gentamicin is closely related to oxidative stress and damage to kidney cells [78,79]. In this context, the decrease in the frequency of mtDNA damage observed in animals treated with CR may reflect a lower degree of oxidative damage to mitochondria. At the same time, the absence of changes in the number of mtDNA copies suggests that mitochondrial biogenesis did not undergo significant changes under these conditions. We propose that these mitochondrial benefits are coordinated by mTORC1 inhibition, as mTORC1 regulates mitochondrial biogenesis via PGC-1α [80] and mtDNA repair pathways [81], both of which are critical for mitigating gentamicin-induced mitochondrial damage. Taken together, these data indicate that CR is associated with changes in redox parameters and mtDNA damage in renal tissue, leading to improved mitochondrial stability and possibly reflecting improved mitochondrial quality control mechanisms [82,83,84] or activation of specific antioxidant protection signaling pathways.

Despite these findings, several limitations of the present study should be acknowledged. First, the assessment of autophagy was based on LC3 processing and did not include direct evaluation of autophagic flux. Second, mitochondrial function was not directly measured, and conclusions are based on mtDNA-related parameters. Third, the sample size was relatively small, which may affect statistical power. Finally, the study design does not allow the establishment of causal relationships between the observed molecular changes and renal protection. Future studies incorporating functional assays and interventional approaches are needed to further elucidate the underlying mechanisms.

## 5. Conclusions

**Figure 7:**
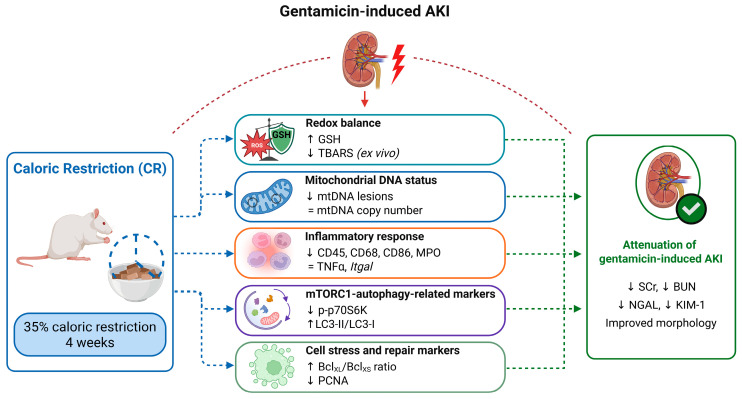
Schematic summary of CR-mediated effects in gentamicin-induced AKI. CR was associated with changes in mTORC1-autophagy signaling, improved redox parameters, reduced mitochondrial DNA damage, and modulated inflammatory responses. These beneficial changes were accompanied by altered pro-apoptotic signaling through the Bcl-XL/Bcl-XS balance, resulting in reduced kidney damage and restored renal function. Created in BioRender. Buyan, M. (2026), https://BioRender.com/dstj5bk (accessed on 7 May 2026).

This study demonstrates that short-term CR provides substantial protection against acute kidney injury induced by gentamicin in rats. The renoprotective effect of CR was associated with increased antioxidant activity, lowered mitochondrial DNA damage, reduced inflammatory infiltration, and modulation of nutrient-sensing pathways related to autophagy (Figure 7). Rather than targeting a single molecular cascade, CR appears to induce complex metabolic and redox adaptations that are associated with enhanced renal resistance to injury. These findings suggest that CR may represent a promising non-pharmacological strategy for reducing drug-induced nephrotoxicity. Since the identified molecular changes are associative and based on static markers, future studies using dynamic functional analyses, interventional approaches, and clinically relevant models are required to further clarify the underlying mechanisms and to evaluate the translational potential of CR or its mimetics in preventing acute kidney injury.

## Figures and Tables

**Table 1:** Primer sequences for measurement of gene expression.

Gene Name	Sequence of Forward Primer	Sequence of Reverse Primer
*Gapdh*	GGCTCCCTAGGCCCCTCCTG	TCCCAACTCGGCCCCCAACA
*Itgal*	TGGCAGATGTGGTTGTAGG	TCTGGAAGCACACCTTGAG
*Tnf*	ATGGGCTCCCTCTCATCAGT	GCTTGGTGGTTTGCTACGAC

## Data Availability

The datasets generated and analyzed during the current study are available from the corresponding author on reasonable request.

## Abbreviations

The following abbreviations are used in this manuscript:

Bcl-X_L_	B-cell lymphoma-extra large
Bcl-X_S_	B-cell lymphoma-extra short
BUN	blood urea nitrogen
GSH	reduced glutathione
GPx	glutathione peroxidase
KIM-1	kidney injury molecule-1
LC3	microtubule-associated protein 1A/1B-light chain 3
mTOR	mammalian target of rapamycin
MPO	myeloperoxidase
NGAL	neutrophil gelatinase-associated lipocalin
ROS	reactive oxygen species
SCr	serum creatinine
